# Different Pathological Roles of Toll-Like Receptor 9 on Mucosal B Cells and Dendritic Cells in Murine IgA Nephropathy

**DOI:** 10.1155/2011/819646

**Published:** 2011-06-30

**Authors:** Tadahiro Kajiyama, Yusuke Suzuki, Masao Kihara, Hitoshi Suzuki, Satoshi Horikoshi, Yasuhiko Tomino

**Affiliations:** Division of Nephrology, Department of Internal Medicine, Faculty of Medicine, Juntendo University, Tokyo 113-8421, Japan

## Abstract

Although pathogenesis of IgA nephropathy (IgAN) is still obscure, pathological contribution of mucosal immunity including production of nephritogenic IgA and IgA immune complex (IC) has been discussed. We have reported that mucosal toll-like receptor (TLR)-9 is involved in the pathogenesis of human and murine IgAN. However, cell-type expressing TLR9 in mucosa remains unclear. To address this, we nasally challenged cell-specific CpG DNA ((i): dendritic cell: (DC), (ii): B cell, (iii): both), known as ligand for TLR9, to IgAN prone mice and analyzed disease phenotype of each group. After 8 times of the weekly administration, every group showed deterioration of glomerular damage. However, CpG-A-group showed clear extension of mesangial proliferative lesions with increase of serum IgA-IgG2a IC and its glomerular depositions, while CpG-B-group showed extent of glomerular sclerotic lesions with increase of serum and glomerular IgA and M2 macrophage infiltration. Present results indicate that mucosal TLR9 on B cells and DC may differently contribute to the progression of this disease via induction of nephritogenic IgA or IgA-IgG IC, respectively. This picture is suggestive for the pathological difference between child and adult IgAN.

## 1. Introduction

Although the definition of IgA nephropathy (IgAN) is simple [1], the disease shows wide variation in clinical course and pathological phenotypes, both of which occur independent of disease duration after its onset. The clinical and pathological manifestations of IgAN also vary between adults and children [2–4]. The pathological factors that are the main determinants for this heterogeneity have not been elucidated to date.

Clinical evidence from kidney transplantation strongly indicates that IgAN pathogenesis is primarily linked to abnormalities in the systemic IgA immune system, rather than to intrinsic abnormalities in renal cells [5–8]. Previous reports have demonstrated that mesangial and serum IgA1 show abnormal O-glycosylation in IgAN cases [9, 10]. In this regard, the contribution of galactose-deficient IgA1 (GdIgA1) and glycan-specific anti-IgA IgG antibodies has recently been implicated in the pathogenesis of IgAN [11–14]. However, the underlying mechanisms by which these nephritogenic IgA and IgG immune complexes (IC) are produced remain obscure.

Studies on bone marrow (BM) or BM transplantation in IgAN [15–18] suggest that nephritogenic IgA is overproduced in systemic immune sites, such as BM. There is also clinical evidence that episodic macrohematuria coincides with mucosal infections [19], abnormal responses to mucosal vaccination [20, 21], and tonsillectomy in IgAN patients with long-term renal survival [22]. These findings indicate that dysregulation of the mucosal immune system is involved in the pathogenesis of IgAN [23]. On the basis of the findings of an elegant series of studies carried out in the 1980s, van Es et al. hypothesized that a “mucosa-BM axis” exists in IgAN. This axis was thought to be involved in continual trafficking of cells between mucosal sites and BM in the IgA immune system [24, 25]. Clinical and experimental studies in the last decade have revealed the detailed mechanisms by which lymphocytes travel between the mucosa and systemic lymphoid tissues. Although these findings support the hypothesis proposed by van Es et al., the cell types involved and their contribution to the immune system remain unclear [26].

Recently, we carried out experimental and clinical studies [27, 28] which demonstrated that toll-like receptor 9 (TLR9) is a key participating molecule in innate and mucosal immunity, and that it has a pathological role in both human and murine IgAN. These studies also showed that the activation of TLR9 on mucosal sites, particularly mucosal sites in the upper respiratory tract, was important for the progression of IgAN. These findings therefore provide clear evidence that the cells responsible for expressing TLR9 may be localized on mucosal sites, including the tonsils. TLR9 is expressed mainly by B cells and dendritic cells (DCs) [29–31], and as both these cells play key roles in innate/mucosal immunity, it is possible that the activation of TLR9 in the mucosa may be involved in the pathogenesis of IgAN. However, the contribution of each cell to the pathogenesis via TLR9 activation has not been examined. The innate immune system of vertebrates is able to distinguish self-DNA from bacterial or other prokaryotic DNA. This is achieved by detecting unmethylated CpG-oligonucleotides (ODNs), in particular base contexts “CpG motifs,” via pattern recognition receptors, such as TLR9 [32–35]. Different CpG-ODNs have been used to study cell regulation by TLR9 in DC, and it has been shown that CpG-A-ODN induces large amounts of IFN-*α* in plasmacytoid DC, CpG-B-ODN acts as a potent stimulant of B cells, and CpG-C-ODN functions as an activator of both B cells and DC [36–38]. However, the detailed regulatory mechanisms in specific cell types has yet to be established [39]. In the present study, we examined the role of TLR9 on each cell type involved in IgAN pathogenesis, by administering cell-specific TLR9 ligands to a recently established IgAN-prone mouse model [40, 41].

## 2. Material and Methods

### 2.1. Animals

The ddY mice (SLC Japan, Shizuoka, Japan) were maintained in a specific pathogen-free room at the animal facility of Juntendo University Faculty of Medicine and provided with regular chow (MF; Oriented Yeast, Tokyo, Japan). The original ddY mice were maintained as outbred animals and were therefore genetically heterogeneous. However, we evaluated their renal histology by serial biopsies and found that they could be classified into three groups on the basis of their renal lesions. Approximately 35% of these mice had glomerulonephritis with mesangial IgA deposition before 20 weeks of age [42]. We defined these mice as early-onset mice and subsequently carried out inbreeding of the group over 20 generations. The incidence rate of IgAN onset in the IgAN-prone mice was almost 100%. We used these IgAN-prone mice under specific pathogen-free conditions, with age-matched BALB/c as controls (*N* = 3). The experimental protocol was approved by the Ethics Review Committee for Animal Experimentation at Juntendo University Faculty of Medicine.

### 2.2. Immunization with CpG-ODN

For assessment of the exogenous pathogen-mediated immune response, three different types of CpG-ODN were administered nasally. The administered CpG-ODN included three types, A, B, and C, based on the type of effector cell targeted. Type A activated DC, type B activated B cells, and type C stimulated both DC and B cells [36–38]. In the present study, ODN1585 (Invivogen, San Diego, California, USA), ODN1668, and ODN2395 were used as types A, B, and C CpG-ODN, respectively. Four-week old IgAN-prone ddY mice were challenged nasally with 10 *μ*g of each CpG-ODN (*N* = 3) at weekly intervals for eight consecutive weeks. We also prepared ddY mice nasally challenged with CpG-ODN-vehicle alone (endotoxin free water) as vehicle control (*N* = 3). For evaluation of renal damage, the same dose of CpG-ODN was administered to age-matched BALB/c mice (*N* = 3). Blood and urine samples were collected before and after each administration. After eight weeks from the first mucosal immunization, kidney samples were collected for histopathological evaluation.

### 2.3. Histology and Immunohistochemistry

The kidney samples were fixed in 10% neutral phosphate-buffered formalin, embedded in paraffin, followed by the preparation of 2 *μ*m thick sections. The sections were then stained with periodic acid-Schiff reagent or Azan for assessment of histological changes by light microscopy. For immunohistochemical analysis, snap-frozen 3 *μ*m thick renal sections were stained with goat anti-mouse IgA antibody (Bethyl, Montgomery, TX, USA) or rabbit anti-mouse IgG antibody (Rockland, Philadelphia, Pennsylvania, USA). DyLight 488 conjugated anti-goat IgG antibody (Rockland) or DyLight 649 conjugated anti-rabbit IgG antibody (Rockland) were used as secondary antibodies. Frozen kidney sections fixed in acetone were stained with the following antibodies: type I collagen (Abcam, Cambridge, UK), type IV collagen (Abcam), *α*-smooth muscle actin (*α*-SMA; Dako, Glostrup, Denmark), CD68 (AbD Serotec, Raleigh, USA), and CD204 (AbD Serotec). Horseradish peroxidase-labeled goat anti-rat IgG antibody (Nichirei Bioscience, Tokyo, Japan) or horseradish peroxidase-labeled goat anti-rabbit IgG antibody (Dako) were then applied as the secondary antibodies. Bound antibodies were detected using an enhanced DAB kit (Dako). Kidney specimens that contained more than 30 glomeruli were used for the histopathological analysis, with the degree of fluorescence intensity being evaluated using an Olympus FV1000 system (Olympus, Tokyo, Japan) and Photoshop Element 2.0 (Abode, San Jose, USA). The degree of mesangium matrix expansion was evaluated using the Carl Zeiss Axioskop2 plus and Carl Zeiss KS 400 3.0 systems (Carl Zeiss, Oberkochen, Germany).

### 2.4. Serum and Urinary Analyses

Blood samples were obtained from the orbital venous plexus using capillary tubes. Serum IgA and IgG levels were measured using an ELISA kit (IgA or IgG Quantitation, Bethyl). Serum IgA-IgG2a IC levels were determined by sandwich ELISA, as reported previously [43]. Urinary albumin was also measured using an ELISA kit (Albuwell, Exocell, Philadelphia, Pennsylvania, USA).

### 2.5. Statistical Analysis

The correlation between different parameters in the groups was analyzed by ANOVA. Data are expressed as mean ± SD or median values. *P* < .05 was considered statistically significant. All the statistical analyses were performed using the Windows version of StatView 5.0 software (Abacus Concepts, Berkeley, CA, USA).

## 3. Results

Each CpG-ODN exacerbated pathologically different glomerular lesions in murine IgAN models.

**Figure 1:**
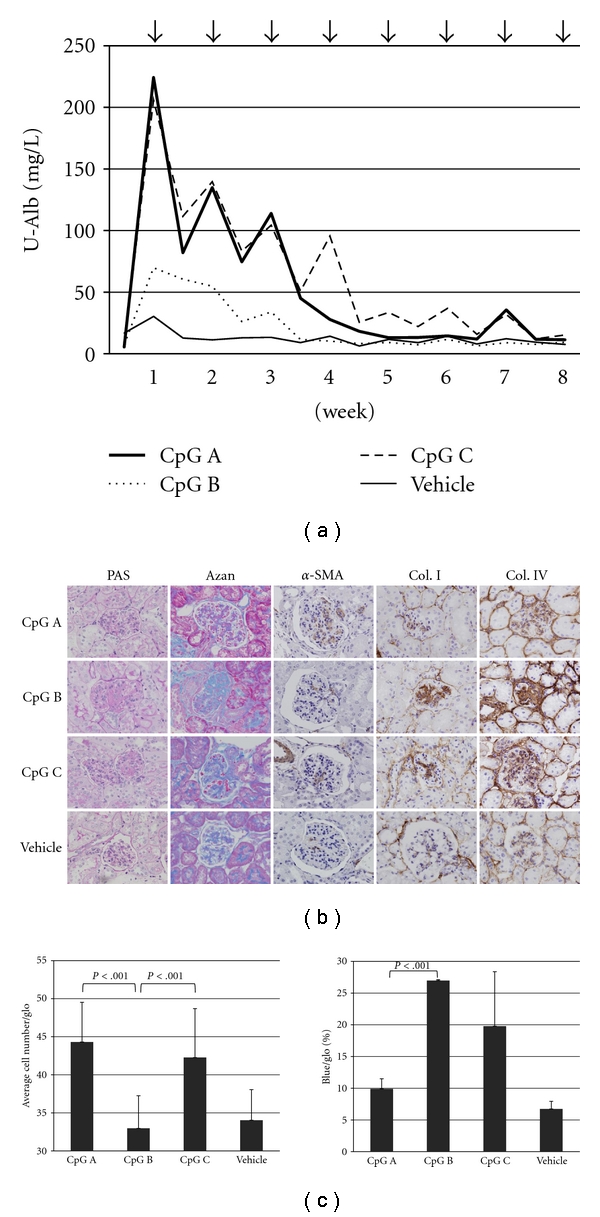
The disease course in each CpG-ODN group. (a) Urinary albumin in each group. Arrows indicate administration of each CpG-ODN agent. Urine samples were collected before and after administration. (b) Glomerular pathological changes at eight weeks. Glomerular damage in each CpG-ODN and vehicle control group were evaluated by PAS and Azan staining, and immunohistochemical analysis of *α*-SMA, type I collagen (Col. I), and type IV collagen (Col. IV). (c) Evaluation of cell proliferation and extracellular matrix (ECM) expansion in the glomeruli of each CpG-ODN group. Cell proliferation was evaluated by counting the average cell number in each glomerulus. ECM expansion was evaluated by the percentage of blue areas in each glomerulus stained by AZAN. In both experiments, more than 30 glomeruli were evaluated in each mouse. Magnification in (b): 400X.

As reported previously [44], CpG-ODN administration aggravated urinary albumin and glomerular injury in IgAN-prone mice, in comparison with the aggravation in the ddY vehicle control group (Figure 1). On the other hand, CpG-ODN administration caused no elevation in urinary albumin excretion and/or glomerular damage in normal Balb/c mice (data not shown). However, each dose of CpG-ODN induced a different amplitude of aggravation in urinary albumin excretion (Figure 1(a)), and also induced different types of pathological lesions (Figure 1(b)). CpG-A-ODN administration induced more proliferative glomerular lesions and higher urinary albumin excretion, while CpG-B-ODN induced greater ECM expansion in glomeruli, as shown by staining with Azan and collagen types I and IV (Figure 1(b)). CpG-C-ODN administration induced a mixed type of damage. Semiquantification of the staining patterns by counting cell numbers and calculating the ratio of ECM area in the glomeruli confirmed the presence of significant differences in the pathological lesions (Figure 1(c)). Activation of TLR9 on DC and B cells in mucosal sites caused different aggravation of glomerular damage in murine IgAN, a finding that highlighted the different roles of these cells in the disorder.

Each CpG-ODN may have a different capacity to induce nephritogenic IgA and IgA-IgG2a IC.

**Figure 2:**
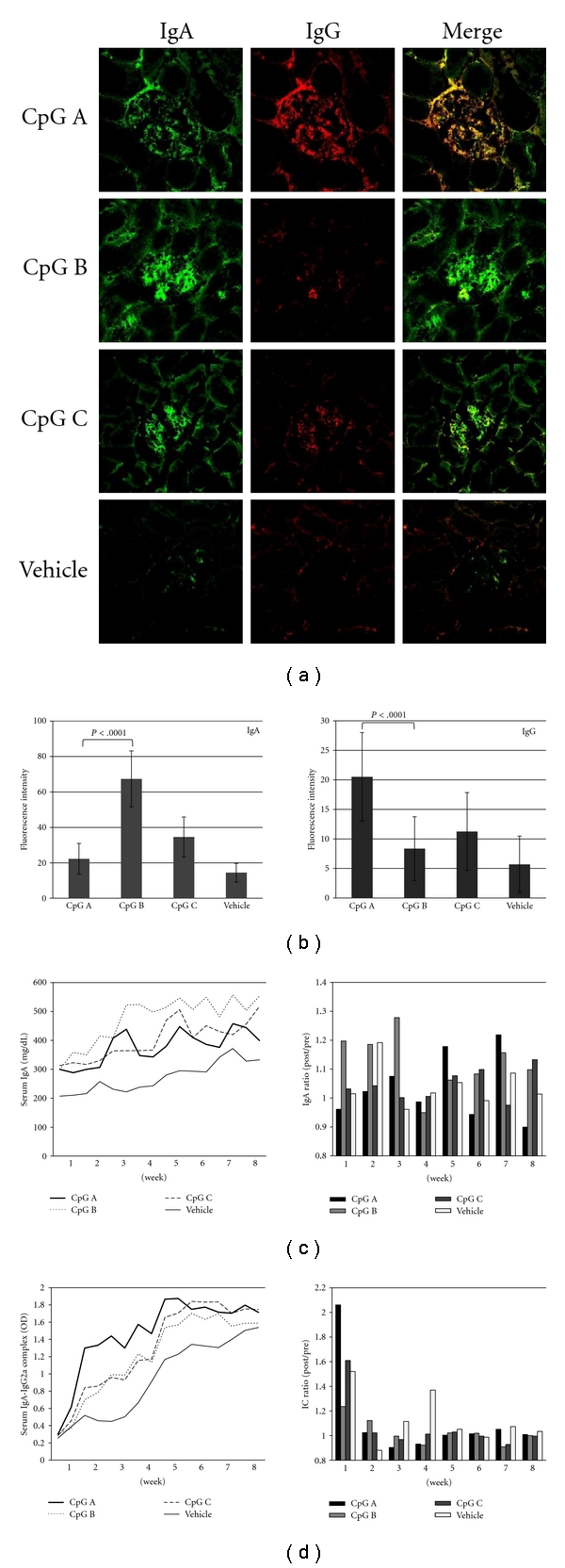
Glomerular deposition and serum levels of IgA, IgG, and IgA/IgG immune complexes (IC). (a) Immunofluorescence staining of IgA and IgG in the glomeruli of each CpG-ODN group. (b) Fluorescence intensity of IgA and IgG in glomeruli. The fluorescence intensity of each immunoglobulin was evaluated in more than 30 glomeruli. (c) Serum IgA (mg/dL) and (d) IgA-IgG2a IC (OD). The ratios of pre/post administration for IgA and IgA-IgG2a IC were also evaluated. Magnification in (a): 400X.

We have previously reported that this model of IgAN-prone mice have codeposition of IgA and IgG in their glomeruli, similar to that seen in human IgAN [42], and that the severity of this disease correlates closely with serum IgG2a levels [43]. On the basis of these findings we examined the glomerular deposition of IgA and IgG and the serum levels of IgA and IgA-IgG2a IC. The fluorescence intensity of glomerular IgA in the CpG-B-ODN group was significantly higher than that in the CpG-A-ODN group (Figure 2(a)). In contrast, the CpG-A-ODN group showed a significantly higher intensity of IgG than the CpG-B-ODN group (Figure 2(b)). The CpG-B-ODN and CpG-A-ODN groups also showed consistently higher serum levels of IgA and IgA-IgG2a IC, respectively (Figure 2(c)). The ratio of pre- and postadministration serum IgA levels was higher in the CpG-B-ODN group until 8 weeks, while the ratio of the pre- and postadministration serum IgA-IgG2a IC levels during the early phase were higher in the CpG-A-ODN and CpG-C-ODN groups (Figure 2(d)).

Polarization of glomerular macrophages differs in each CpG-ODN group.

**Figure 3:**
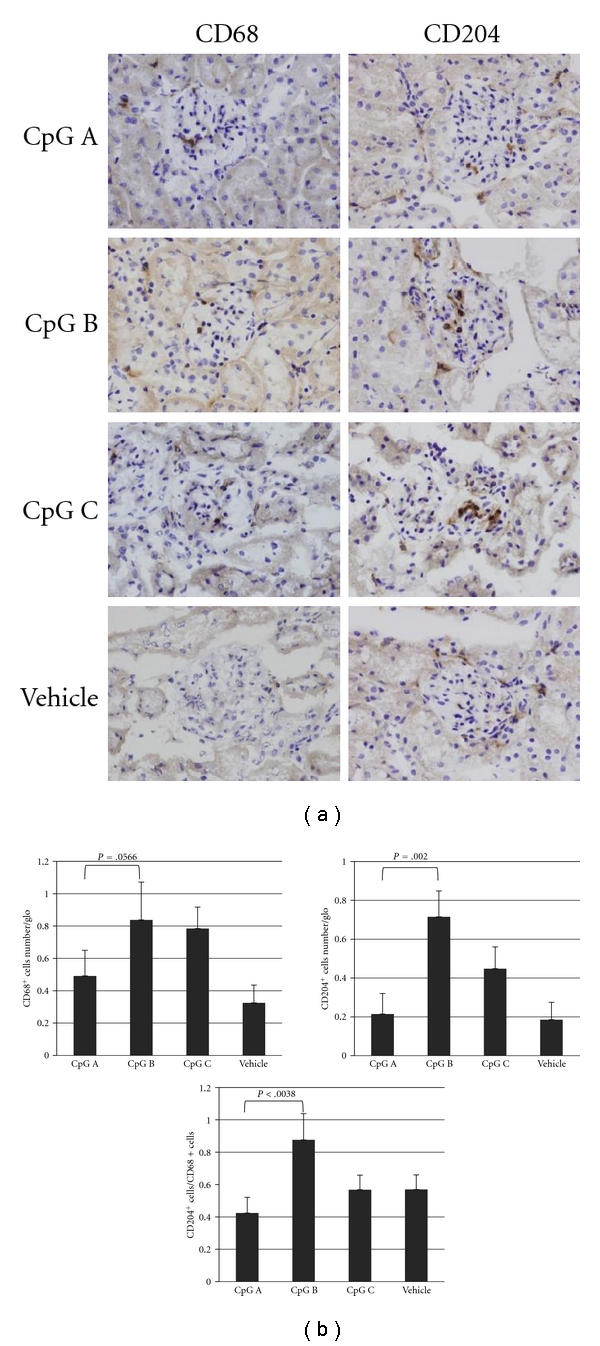
Phenotype of infiltrated glomerular macrophages in each CpG-ODN group. The phenotypes of infiltrated macrophages were evaluated immunohistologically using anti-CD68^+^ (pan macrophage) and anti-CD204^+^ (M2 macrophage) antibodies (a). In addition, the average number of each infiltrated glomerular macrophage and the phenotypic ratio were also evaluated (b). More than 30 glomeruli were evaluated in each stained section from each animal. Magnification in (a): 400X.

Previous studies have suggested that polarization of glomerular macrophages may contribute to the patterns of glomerular damage in IC-mediated glomerulonephritis, including IgAN [45–47]. Accordingly, we examined the expressions of CD68 (pan macrophages) and CD204 (M2 macrophages) in the glomerular macrophages (Figures 3(a) and 3(b)). As shown in Figure 3(b), the number of CD204^+^ cells increased in the CpG-B-ODN group. The CD204^+^/CD68^+^ ratio in glomeruli was greater than 80% in the CpG-B-ODN group and less than 50% in the CpG-A-ODN group. This finding suggests that macrophages in the CpG-B-ODN group were polarized mainly to M2.

## 4. Discussion

The findings of the present study revealed that CpG-A-ODN and CpG-B-ODN exacerbated different types of glomerular damage. This damage consisted mainly of mesangial proliferation or ECM expansion in association with serum elevation of IgA-IgG2a IC and enhanced glomerular deposition of IgA. The study therefore provides further confirmation that activation of mucosal TLR9 by CpG-ODN aggravates murine IgAN [40] and strongly indicates that the activation of TLR9 on DC and B cells may afford differing contribution to the disease process.

It is widely accepted that mesangial IgA is predominantly IgA1 that displays abnormal O-glycosylation [9, 10] in human IgAN. This indicates that aberrantly glycosylated IgA has a pathological role in human IgAN [48, 49]. It is also well known that tonsillar B lymphocytes in IgAN patients produce under-O-glycosylated IgA1 such as GdIgA1 [50–52]. IgAN may be associated with a maldistribution of nephritogenic IgA secreting B cells from mucosal to systemic sites including bone marrows [17, 23, 53]. Since TLR directly contribute to homing process of lymphocytes and DC [54–56], mucosal/tonsillar B cells secreting the aberrantly glycosylated IgA may further disseminate to other lymphoid tissues after TLR activation. In addition, these tonsillar B cells show downregulation of *β* 1,3-galactosyltransferase activity, a key enzyme that catalyzes O-galactosylation at the hinge region of IgA1 [57]. Further, there is evidence suggesting that aberrantly glycosylated IgA is involved in the pathogenesis of murine IgAN [58, 59]. Humans have two isotypes of IgA, IgA1, and IgA2. Specifically, IgA1 produces longer hinge lesions with O-glycosylation sites, whereas murine IgA does not [49, 60]. This implies that GdIgA1 may not be involved in the pathogenesis of murine IgAN. However, recent evidence suggests that aberrant glycosylation of N-glycans regulated by *β* 1,4-galactosyltransferase are involved in the pathogenesis of murine IgAN [58, 59]. It is therefore possible that aberrant modifications of carbohydrates in serum IgA are involved in the development of both human and murine IgAN, independent of whether or not the carbohydrates are O- or N-glycans. The IgAN-prone mice used in the present study were originally established from ddY mice [42] which produce nephritogenic aberrantly glycosylated IgA [59]. Our recent study also indicated that B cells are responsible for murine IgAN producing nephritogenic IgA in T-cell-independent (unpublished data) and germinal center-independent manners [61]. In addition to exacerbating murine IgAN by increasing serum IgA levels and glomerular deposition of CpG-B-ODN, mucosal B cells responsible for these actions may also cause increased production of aberrantly glycosylated nephritogenic IgA via activation of their own TLR9.

It is now apparent that serum levels of GdIgA1 are often elevated in IgAN patients [11–13, 62]. Gharavi et al. recently reported that GdIgA1 levels were increased in 78% of patients with sporadic IgAN and in 25% of their blood relatives, although the majority of relatives with abnormal IgA1 glycoforms were asymptomatic [63]. This finding suggests that additional cofactors are required for development of IgAN in certain cases. In a recent publication Suzuki and coworkers described the characteristics of IgG autoantibodies to abnormally glycosylated IgA1 secreted by immobilized B cells derived from patients with sporadic IgAN [13]. The serum levels of these IgG autoantibodies correlated closely with the degree of proteinuria, suggesting that IC formation of aberrantly glycosylated IgA and glycan-specific IgG antibodies may be an additional cofactor required for full development of the disease [53, 64]. In this regard, it is noteworthy that the serum levels of IgA-IgG2a IC, but not of IgA, were shown to correlate closely with the severity of glomerular lesions in IgAN-prone mice [43]. In addition to the aberrant glycosylation of IgA [58, 59], similar mechanisms about immune complex formation may underlie the progression of both human [13] and murine [43] IgAN. Therefore, disease aggravation caused by elevation of serum IgA-IgG2a IC levels and the increased codeposition of IgA and IgG observed in the CpG-A-ODN group, suggest that mucosal TLR9 activation on DC may be involved in the production of glycan-specific autoantibodies. Recent clinical and experimental papers have indicated that abnormal somatic mutations in the germinal center may contribute to the production of autoantibodies in autoimmune diseases [56, 65]. These papers also suggested that DC-B-cell interactions may be involved in such T-cell-independent antibody production, presumably including polyreactive and autoreactive antibodies. Therefore, DC-B-cell interactions should be examined carefully in future studies investigating the pathogenesis of IgAN.

The present models treated with each CpG-ODN highlighted the fact that the two major pathological characteristics of IgAN, mesangial proliferation and ECM expansion, may combine to result in an increase in serum levels of IgA-IgG2a IC and IgA in glomeruli. It is known that heat-aggregated IgA has strong capacity to induce mesangial proliferation, and ECM expansion via cytokine production such as IL-6 and TGF-*β* [66, 67]. However, recent papers showed that human aberrantly glycosylated IgA has no strong capacity for mesangial proliferation, while IgG-conatining IgA IC shows strong capacity for the proliferation [68, 69]. Therefore, same mechanisms for mesangial activation by IgA or IgA-IgG IC may underlie murine IgAN. Interestingly, these two characteristics represent the typical pathological manifestations of child and adult IgAN. Further, it is known that mesangial enlargement in pediatric IgAN is due mainly to mesangial hypercellularity rather than to increased production of matrix, whereas mesangial matrix expansion is the predominant finding in adult IgAN [2–4]. This tendency has also been observed in new-onset pediatric and adult IgAN [2]. Although there is still no clear explanation for this mechanism, the roles of macrophage subsets have been considered [70]. Macrophages have been implicated as mediators of renal injury in glomerulonephritis and the degree of their accumulation is predictive of disease progression in IgAN [70]. Blood monocytes differentiate into macrophages, the phenotype of which is dependent upon the local microenvironment encountered. These polarized macrophages have been classified into two broad groups; classically activated (M1) or alternatively activated (M2) macrophages [46]. In general, classically activated M1 macrophages are tissue injury type macrophages involved in the expansion of inflammation, while M2 macrophages have immunoregulatory and immunosuppressive functions [45, 46]. While some studies have argued a tissue reparative role for M2 macrophages, other studies have suggested that some subtypes of alternatively activated M2 macrophages have a profibrotic phenotype [45, 47, 71]. Indeed, M1 macrophages express CCL15, CCL20, and CXCL9-11 which are upregulated by acute inflammatory cytokines such as IFN*γ* [72, 73]. On the other hand, CCL17, CCL18, and DCIR on M2 macrophages are mainly regulated by anti-inflammatory Th2 cytokines, such as IL-4 [72, 73]. In addition, M1 macrophages strongly express Fc*γ*RI and III known as inflammatory Fc*γ*Rs, while M2 macrophages mainly express Fc*γ*RII known as inhibitory Fc*γ*R [72, 73]. Therefore, increase of IgG-containing IgA IC deposition in glomerulus may influence glomerular cytokine condition and thus subsequent polar form of macrophages, partly via chemokine receptor expressions.

An important finding in the present study was that the majority of glomerular macrophages in the CpG-B-ODN group were CD204^+^ M2 macrophages and were associated with ECM expansion. As M2 or M2-like phenotypic macrophages are increased in adult IgAN [2, 47], it needs to be examined whether the mechanism by which an acute increase in deposition of aberrantly glycosylated IgA in glomeruli induces the activation of M2 macrophages and subsequent fibrotic changes. On the other hand, we found the ratio of M1/M2 macrophages was increased in the CpG-A-ODN group, suggesting that codeposition of IgG delivered by IgA-IgG2a IC may establish an inflammatory milieu for M1 polarization and subsequent further localized elevation of inflammatory cytokines that induce mesangial proliferation, such as IL-6 [66, 74–76].

## 5. Conclusion

The present study demonstrated that mucosal activation of TLR9 on B cells and DC had different contributions to the progression of murine IgAN, presumably via formation of nephritogenic IgA and IgA-IgG IC, respectively. Although further study is necessary to determine how IgA and IC induce pathological manifestation via assembly of M1/M2 macrophage, the present results may provide important clues for future radical treatment modalities for IgAN.
